# War Memories at Winchester

**Published:** 1920-02-21

**Authors:** 


### War Memories at Winchester.

### Royal Hampshire County Hospital.

At the beginning of August 1914 the hospital was running its orderly course, with many of its nurses taking their summer holidays. Those who were away hurried back; those whose holidays were yet due abandoned them; stores of surgical dressings were ordered; bed space was compressed; applications for instruction in nursing poured in. A preliminary offer of thirty beds was made to the War Office, and the first to occupy these were Belgians. At the request of the War Office thirty more beds were shortly added, but the limit of expansion was now reached, for the wards were packed. The number of civilian patients was never lessened. Wooden huts were built on the south side of the main building, under the joint administration of the hospital and the Red Cross Society. Seventy-five beds were thus added, and subsequently another large hut to hold about eighty. Thus 243 soldiers were ultimately cared for within the precincts of the hospital.

The difficulties of cooking and washing had to be met by adaptation of our existing plant; the labours of all the staff were more than doubled.

Some idea of the work performed can be realised from the following statistics: 3,765 wounded soldiers passed through; 850 operations were performed; 4,850 cases of our own and from the military hospital received *x*-ray treatment; 686 cases received massage; 52 nurses were called away for active service; and our monthly bills rose from an average of £600 to £2,000. The strain was met; nothing broke. Good-will and cheerfulness—greatly aided by happy gratitude of the wounded soldiers—reigned everywhere. There was little outward sign of the weight which was being sustained.

The grants from the War Office paid the food bills, but lavish private generosity was needed to make both ends meet, and nobly was it offered. If one may mention names where so many helped, warmest thanks are due to Miss Burrel, to Lady Portal, Mr. Mills, and many others.

For its size this hospital claims to have been second to none, and in the grouping of Red Cross huts round a central building perhaps gave a lead to others.

